# Top-view characterization of broiler walking ability and leg health using computer vision

**DOI:** 10.1016/j.psj.2024.104724

**Published:** 2024-12-22

**Authors:** István Fodor, Marjaneh Taghavi, Esther D. Ellen, Malou van der Sluis

**Affiliations:** Animal Breeding and Genomics, Wageningen University & Research, 6700 AH Wageningen, the Netherlands

**Keywords:** Chicken, Gait, Automation, Phenotyping, Deep learning

## Abstract

Impaired walking ability and leg health are commonly seen in broilers and can negatively impact their welfare. Commonly, walking ability and leg health are assessed manually, but this is time consuming and can be subjective. Automated approaches for scoring walking ability and leg health at the individual level could therefore have great added value. Here, we studied whether automatically extracted top-view walking features of broilers can be used as a proxy for walking ability and leg health. Top-view videos were collected of unmarked broilers walking through a corridor that was placed inside the home pen. From these videos, four top-view features were derived: 1) lateral body oscillation, calculated as deviations from the movement trajectory of the bird, 2) step count, 3) completion time, and 4) length-width ratio of the virtual bounding box encapsulating the bird while walking as an indicator of wing support. We assessed the relationship of these computer vision-based features with manual gait and leg health scores, including hock burn (**HB**) and footpad dermatitis (**FPD**). We observed that birds with worse gait scores (**GS**) had longer completion times, higher step counts and a trend for higher lateral body oscillation levels in the walkway setup. Unsupervised clustering using the K-means algorithm with these walking features showed potential to distinguish birds with GS3+, although differentiating between GS1 and GS2 proved more challenging. We concluded that the length-width ratio of the bounding box during walking was not a suitable proxy for poor gait. We found no relationship between top-view walking features and mild cases of HB and FPD in broilers. Overall, the results of this study indicate that top-view video recordings can provide insight into birds’ walking ability, using features related to movement speed, step count and lateral body oscillation, making automated scoring more feasible on a larger scale in practice. However, these top view features provide little information about mild HB and FPD.

## Introduction

Impaired walking ability is commonly seen in broilers and can cause behavioural changes and welfare problems, as well as economic losses (Knowles et al., 2008; Gocsik et al., 2017; Riber et al., 2021). Commonly, walking ability is assessed visually, for example using the Bristol gait scoring system (Kestin et al., 1992), which is considered the gold standard for gait scoring in broilers (Fodor et al., 2023). However, this requires experienced personnel to observe and manually score individual birds while walking, which is time-consuming and subjective. Genetic selection for leg health aspects that may be related to impaired walking ability, such as tibial dyschondroplasia or hock burn (**HB**), is possible (Kapell et al., 2012) but requires detailed observations of individual birds’ leg health. Therefore, automated approaches for scoring walking ability and leg health at the individual level could have great added value for broiler breeding programs.

Several studies have examined automated ways of scoring walking ability and leg health in broilers. Regarding walking ability, sensors can for example be attached to birds to measure behavioural proxies for GS, such as activity (van der Sluis et al., 2021). Kinetic sensors, such as pedobarographs or force plates, can also be used to assess several aspects of walking in broilers (Corr et al., 1998, 2007). Alternatively, cameras can be used to record proxies for GS, for example based on activity levels (e.g., Dawkins et al., 2009) or lying behaviour (e.g., Aydin, 2017b), to record different walking features, such as speed, step frequency and length, or lateral body oscillation (e.g., Aydin, 2017a; de Alencar Nääs et al., 2021; Li et al., 2023), or to track keypoints on the birds’ bodies (Nasiri et al., 2022; Fodor et al., 2023). For leg health aspects such as HB or footpad dermatitis (**FPD**), several studies examined automated assessment approaches at the slaughter line (e.g., Vanderhasselt et al., 2013; Louton et al., 2022a, 2022b). However, live scoring would be preferable for phenotyping and welfare monitoring purposes. Top-view video and optical flow approaches based on activity and distribution patterns have been implemented to assess flock-level HB and FPD (e.g., Dawkins et al., 2017; Peña Fernández et al., 2018). However, a downside of these approaches is that they only provide flock-level estimates, whereas individual-level records are required for phenotyping purposes. Overall, there are multiple potential approaches that can aid in automated gait and leg health scoring, yet scaling these up for high-throughput phenotyping is still challenging.

In earlier work, we used back-view video data of broilers walking individually in a corridor and applied a pose estimation model to study different walking characteristics in broilers (Fodor et al., 2023). We observed that broilers with good versus suboptimal gait showed differences in hock joint lateral angles, hock-feet distance ratios and relative step height. However, back-view videos of broilers for pose estimation are challenging to obtain under practical conditions. Back-view pose estimation requires a clear view of individual birds walking, but birds are commonly housed in large groups, which can lead to occlusion by other birds. Top-view video recordings might be more feasible to obtain and are less prone to occlusion by other birds.

In this study, top-view videos were collected of unmarked broilers walking through a corridor that was placed inside the home pen. From these videos, several top-view features were derived, including 1) lateral body oscillation, calculated as deviations from the movement trajectory of the bird, 2) step count, 3) completion time, and 4) length-width ratio of the virtual bounding box encapsulating the bird while walking as an indicator of wing support. Subsequently, it was assessed whether and how these features are associated with manually determined GS and contact dermatitis-related leg health scores, namely HB and FPD. Overall, this study provides insights into the utility of top-view recorded proxies for individual-level walking ability and leg health scoring in broilers, making automated scoring more feasible on a larger scale in breeding practice.

## Materials and methods

### Ethical statement

Data were collected under control of Cobb Europe (Boxmeer, the Netherlands). Cobb Europe complies with the Dutch legislation on animal welfare. This study is not considered to be an animal experiment under the Law on Animal Experiments, as confirmed by the local Animal Welfare Body (July 11, 2022, Lelystad, the Netherlands).

### Data collection

**Fig. 1:**
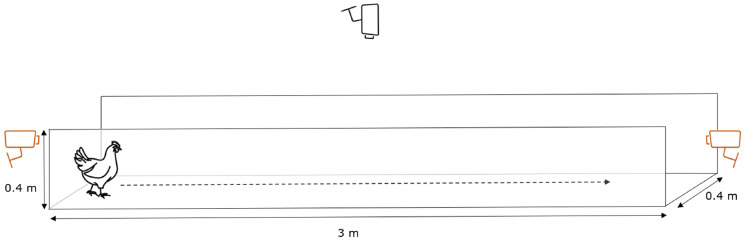
Schematic view of the corridor (cameras displayed in orange were not used in this study).

Video data were collected for 121 tagged 34-day-old broilers (one day prior to slaughter age), representing a mix of commercial genetic lines, walking individually through a corridor. The corridor was approximately 3 m long and 0.4 m wide, and the walls had a height of 0.4 m. The schematic setup of the corridor is shown in Fig. 1**.** The corridor was placed inside the home pen, having the birds’ regular bedding (wood shavings) as floor material. Reolink RLC-510A cameras (Reolink, Hong Kong, China) were placed at the start (back-view; not used in this study), end (front-view; not used in this study) and above (top-view) the corridor, and recorded videos with a resolution of 2560 × 1920 pixels and a framerate of 11 frames per second. These videos were automatically saved as one-hour .mp4 files each and stored on a hard disk. Before the corridor was placed inside the home pen, a large checkerboard printout was moved across the floor of the pen, for camera calibration purposes (see section **Video Processing**).

Birds were individually placed at the start of the corridor and were then able to walk to the end of the corridor, that ended in their home pen. Before being placed in the corridor, birds were individually scored for HB and FPD and their body weights were recorded. HB was scored on a scale from zero (healthy skin on the hock) to five (extensive lesions on the hock skin area, covered with dark crust, hock warm and swollen), with separate scores for the left and right hock. FPD was also scored on a scale from zero (healthy skin on the footpad) to five (extensive lesions covering the entire footpad area, foot swollen and covered with dark crust), with separate scores for the left and right foot. For both the HB and the FPD scores, a mean score was calculated that averaged the value of the left and the right legs, to obtain a single score per bird. The highest HB score on a single foot was 2 (corresponding to a minor red lesion), and only one broiler had HB score 2 on one foot. Due to the low prevalence of HB among the studied broilers, we created a binary class based on the mean HB scores: HB 0 (HB score 0 on both feet), and HB 1 (non-zero HB score on at least one foot). Regarding FPD, only two birds had FPD scores of 4, and nine birds had FPD scores of 3 on at least one of their feet. We defined three groups based on the mean FPD scores: FPD 0 (FPD score 0 on both feet), FPD 1 (mean FPD score of the two feet larger than 0, but smaller than 2), and FPD 2 (mean FPD score of the two feet 2 or higher). While the birds were walking through the corridor, their GS were observed and recorded manually by a single experienced observer, on a scale from zero to five that is described in van der Sluis et al. (2021) and Fodor et al. (2023), and closely resembles the scoring system developed by Kestin et al. (1992). In this scoring system, score 0 represents birds walking very well, score 1 means controlled walk with capability to stand straight, score 2 is a relatively good, oriented walk, with score 3 the bird is more out of balance, can translocate well but sits down quickly, with score 4 the bird walks poorly, with bent or spread legs, waddling, legs pointing outward, wings often hanging down, and with score 5 broilers can barely walk and use their wings for support when walking. There were no birds with GS 0 or 5 in our study, and only two broilers had GS 4. Therefore, GS 3 and GS 4 were merged together for subsequent analyses (GS 3+). If birds did not progress through the corridor, they were gently nudged using a wire-net panel attached to a stick. However, we aimed to limit stimulation of the birds so their walking characteristics reflect voluntary walking as much as possible. It was manually noted at what exact time each bird was placed in the corridor, to allow matching of the videos to specific bird identities.

### Video processing

**Fig. 2:**
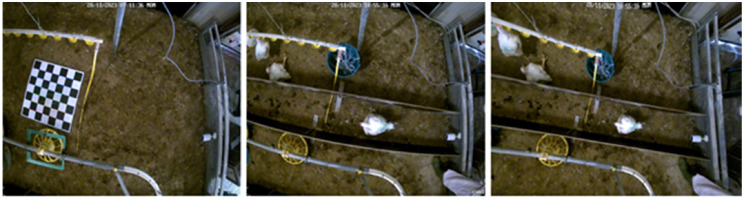
Undistortion processing of the frames, showing the checkerboard for camera calibration (left), an original frame (middle) and the resulting undistorted frame (right).

First, the one-hour duration top-view videos were cut into individual videos per bird, based on the manually determined start and end times of entering and exiting the corridor, and frames were extracted from the videos with a rate of 11 frames per second. Subsequently, we implemented a calibration process, to ensure that the images produced by the camera were geometrically accurate and free from distortions that are typically introduced by the lens. For this, a large checkerboard printout of six by six squares (41.1 × 41.1 cm) was used. The checkerboard was moved across the floor of the pen while top-view video recordings were made. The resulting videos were used to obtain camera parameters that were subsequently applied to correct for camera distortion in the captured frames. Fig. 2 shows the checkerboard, an original frame with distortion and a resulting undistorted frame.

**Fig. 3:**
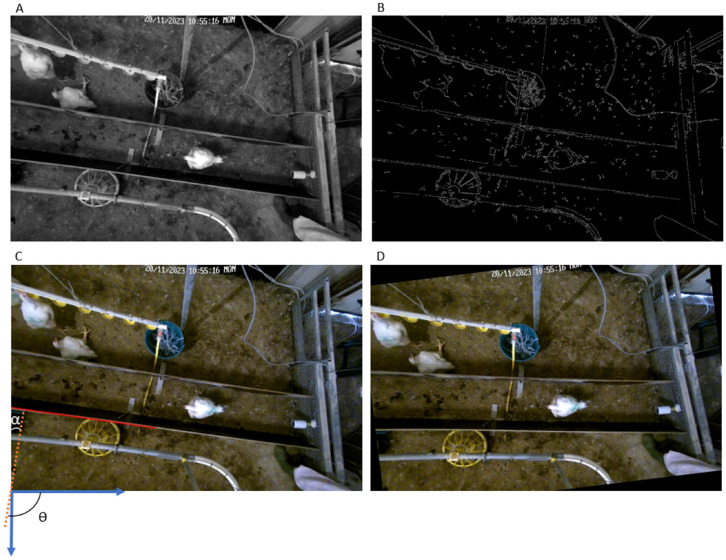
The process of rotating the frames, showing a) the grayscale frame, b) the detected edges, c) the red line and the angle Ɵ are detected by the Hough transform and α is the rotation angle, d) the rotated frame.

Next, the undistorted frames were rotated to align the walkway path horizontally, similar to the approach used by Li et al. (2023). To this end, the original frame was converted into grayscale to simplify the edge detection process (Fig. 3**a**) and edges in the grayscale image were detected using the Canny edge detection algorithm (Canny, 1986; Fig. 3**b**). Then a Hough Line Transformation (Duda and Hart, 1972) was applied to the edge-detected image to find lines and the line on the lower wall of the walkway path was selected as reference (Fig. 3**c**). Using the extracted angle (θ) of the detected line, the image was rotated to align the walkway path horizontally, where the rotation angle was determined by subtracting 90 degrees from the extracted angle (Fig. 3**d**).

### Deep-learning object and pose detection model development

We subsequently detected birds in the walkway, as well as the position of their heads, to be able to track their location and direction (i.e., which way they were facing) over time. Using the Computer Vision Annotation Tool (**CVAT**; v1.1.0 (Sekachev et al., 2020)), 170 frames were annotated for training by a single observer drawing bounding boxes around the bird and annotating the top of the head. An additional 30 frames were used for validation and 50 frames for testing. Then, a pre-trained YOLOv8 pose model (Jocher et al., 2023) was trained and validated for detecting broilers in the walkway and for the detection of the head keypoint. The model was trained for 200 cycles (epochs), processing small batches of 2 examples at a time. To prevent overfitting, we used early stopping. This technique stops the training process if the model's performance on a separate validation set doesn't improve for 50 cycles. Early stopping helps ensure the model doesn't become too specialized to the training data, so it can generalize better to new data. The F1 score (formula 4) and mean Average Precision (**mAP**) (formula 3) were used to evaluate the YOLOv8 model performance. The mAP calculates the average area under the precision-recall curve across all classes. It measures the average precision and recall at different Intersection over Union (IoU) thresholds. A higher mAP indicates better performance in balancing precision and recall. The mAP incorporates the trade-off between precision and recall ((1), (2)) and considers both false positives and false negatives. The mAP50 measures the mean average precision at an IoU threshold of 0.5, while mAP50-95 measures the mean average precision across different thresholds ranging from 0.5 to 0.95. The best model parameter was selected based on the model performance in the validation set.

(1) $$Precision=\frac{True\;Positive}{True\;positive+False\;Positive}$$

(2) $$Recall=\;\frac{True\;Positive}{True\;Positive+False\;Negative}$$

(3) $$mAP=\frac{1}{N}\sum_{k=1}^{N}AP_{k}$$

(4) $$F1=2\;\times \;\frac{Precision\times Recall}{Precision+Recall}$$

In Eq. (3), N is the number of classes and AP_k_ is the average precision of class k. In our specific dataset, where we have only one class, the value of N is 1.

**Fig. 4:**
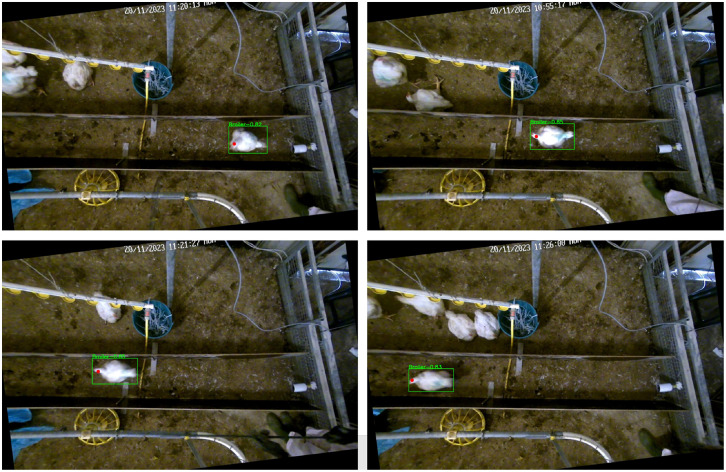
Examples of the automated detection of different broilers.

The algorithm resulted in bounding box and head keypoint coordinates for the birds walking in the corridor. Fig. 4 shows some examples of the detection of the bounding box and head keypoint for different broilers. The video pre-processing steps and YOLOv8 model development were implemented in Python 3.9 (Python Software Foundation, 2022).

### Top-view features

Using the resulting bounding box and head keypoint coordinates, first, the centre point of the bounding box was calculated. Then, it was determined whether birds were facing forward, i.e., if the head keypoint was located towards the end of the walkway relative to the bounding box's centre point. If this was not the case, bounding box and head keypoint coordinates were set to missing, which applied to 0.11 % of the coordinates. If the resulting gap in the sequence of frames was not larger than five frames, the coordinates were imputed using linear interpolation. There were no birds with longer than five frames of consecutive missing values. Each of the time series was subjected to smoothing prior to the quantification of top-view features in order to reduce noise in the data. To this end, each time series was smoothed using the moving average with a window size of three frames. Subsequently, several top-view walking features were derived, including the length-width ratio of the bounding box, completion time, lateral body oscillation, and step count.

The change in bounding box relative proportions (i.e., the length-to-width ratio) was used as a potential indicator of wing support during walking. It was assumed that when a bird uses at least one of its wings during walking, which is indicative of poor gait (Kestin et al., 1992), the width of the bounding box encapsulating the bird suddenly increases, leading to a drop in the length-to-width ratio. Based on manual checks, few occurrences of wing support were observed in the data and the analysis of the bounding box relative proportions as a potential indicator of wing support revealed that changes in these proportions are not unique to wing support. It was difficult to distinguish between changes in bounding box proportions as a consequence of wing support, wing flapping, shaking or turning sideways in the corridor. Due to this low specificity of the bounding box proportion changes and consequent challenging future implementation in practice, we decided not to continue with this indicator.

**Fig. 5:**
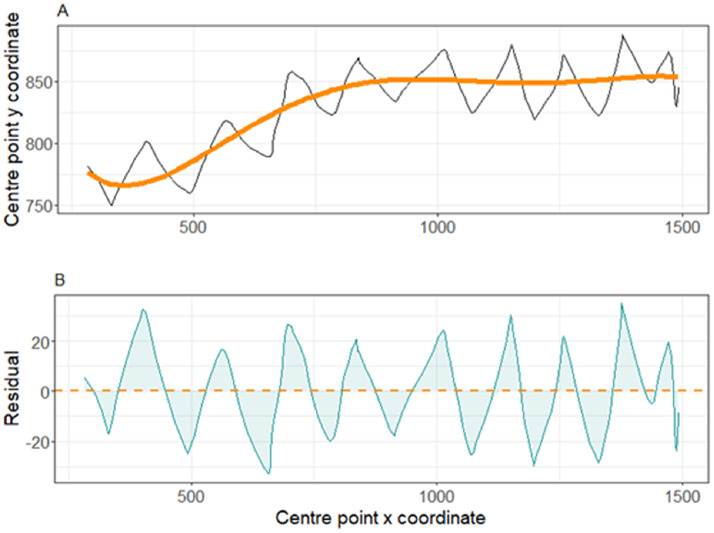
a) tracked path of the bounding box centre point and the estimated movement trajectory of a broiler at 34 days of age; b) the lateral body oscillations of the same broiler during the walkway test.

We used the movements of the bounding box centre point to quantify lateral body oscillation of the broilers. We hypothesized that the movement of the bounding box centre point during the walkway test consists of an overall movement trajectory (which is not necessarily straight even in a walkway) and the actual oscillation around that trajectory. After graphical assessment of several methods (including polynomials of different orders, moving average, and quantile regression), we decided to use a 5th order polynomial to capture the overall movement trajectory of each individual, though the differences among methods were small. After fitting a 5th order polynomial on the bounding box centre point time series of each individual, model residuals were extracted, and used to quantify lateral body oscillation. The mean of the absolute residuals was assigned to each bird to quantify lateral body oscillations. Fig. 5 shows the tracked path of the bounding box centre point of a broiler, the estimated movement trajectory, and the lateral oscillations. When broilers walk, their centre of gravity oscillates around the movement trajectory while moving forward, shifting from left to right and vice versa. Therefore, we estimated step count of each bird as the number of times that the bounding box center point crossed the movement trajectory. Completion time was defined as the time taken (in seconds) by the bird to move from the start to the end of the corridor in the video. One bird (GS 2, HB score 0, FB score 0) did not complete the entire walkway test, therefore, this broiler was removed from further analyses. Another two birds lost their identification tags before the walkway test, therefore, to minimize the chance of errors, these were also excluded. Ultimately, the final dataset included the walking features, gait and leg health scores, and body weight of 118 broilers.

### Statistical analyses

All statistical analyses were performed in R version 4.3.1 (R Core Team, 2023). The relationship among walking features (lateral body oscillation, step count, and completion time) was analyzed using Pearson correlation. The relationship among GS, FPD class, and HB class was analyzed using Fisher's exact test. Differences in body weight by GS and FPD class were analyzed using ANOVA, and the weight difference by HB class was tested using Welch's t-test. The relationship of each walking feature with walking ability (GS class) or leg health (FPD class, HB class) was tested using linear models. The initial model included one of the walking features as the dependent variable, one predictor for walking ability (GS class with 3 levels) or leg health (FPD class with 3 levels, HB class with 2 levels) as a factor, body weight as a covariate, and their two-way interactions. Non-significant (P>0.05) interactions were removed, and the models were refitted. Pairwise comparisons among factor levels were performed using the emmeans package (Lenth, 2023), and P-values were adjusted using Tukey contrasts. The level of significance was set to P≤0.05, and tendency was defined as 0.05<P≤0.10. Furthermore, in order to explore the combined potential of the walking features to assess gait, K-means clustering was performed with all features (lateral body oscillation, step count, and completion time) as input, to identify 3 clusters in the data (i.e., *K* = 3) in an unsupervised way. Then, a confusion matrix was created with the 3 clusters and the 3 GS classes to assess the clusters against GS as a gold standard.

## Results

### Relationship of walking ability, leg health, and body weight

The mean (±SD) body weight of the broilers at 34 days of age was 2291 ± 243 g. Out of the 118 broilers analyzed in our study, 71 (60.2 %) birds had GS 1, 38 (32.2 %) had GS 2, and 9 (7.6 %) were classified as GS 3+. Altogether, 9 broilers (7.6 %) were classified as HB 1, and 109 broilers (92.4 %) as HB 0, that is, more than 90 % of the broilers had zero HB score on both of their legs. Similarly, the majority of the birds had zero FPD scores on both feet, 63 broilers (53.4 %) were classified as FPD 0, 40 broilers (33.9 %) as FPD 1, and 15 broilers (12.7 %) as FPD 2. The body weight, HB score, and FPD score results of the broilers by GS group are shown in Table 1. Birds in higher GS classes had significantly higher body weights than birds with good walking ability (GS 1). In our study, leg health problems did not affect walking ability of the broilers, as we found no relationship between GS class and either HB scores or FPD scores. The proportion of broilers with non-zero HB scores in FPD 0, FPD 1, and FPD 2 was 9.5 %, 5.0 %, and 6.7 %, respectively, and no relationship was found between HB scores and FPD scores (*P* = 0.792). Body weight of broilers did not differ by FPD score (*P* = 0.836). Even though broilers with non-zero HB scores were numerically heavier (HB 0: 2282 ± 244 g, HB 1: 2398 ± 215 g), the difference was not significant (*P* = 0.154).

**Table 1:** Body weight, hock burn (HB) scores and footpad dermatitis (FPD) scores per gait score (GS) category.

Variable	Gait score group			P-value
	GS 1 (n = 71)	GS 2 (n = 38)	GS 3+ (n = 9)	
Body weight (g, SE)	2235 ^a^ (28)	2350 ^b^ (38)	2480 ^b^ (78)	0.003
HB score group (n, %)				
HB 0	64 (90.1)	36 (94.7)	9 (100)	0.647
HB 1	7 (9.9)	2 (5.3)	0	
FPD score group (n, %)				
FPD 0	39 (54.9)	20 (52.6)	4 (44.4)	0.909
FPD 1	24 (33.8)	12 (31.6)	4 (44.4)	
FPD 2	8 (11.3)	6 (15.8)	1 (11.1)	

^a, b^ Different superscript letters indicate significantly different (P≤0.05) body weight

### Model performance

**Fig. 6:**
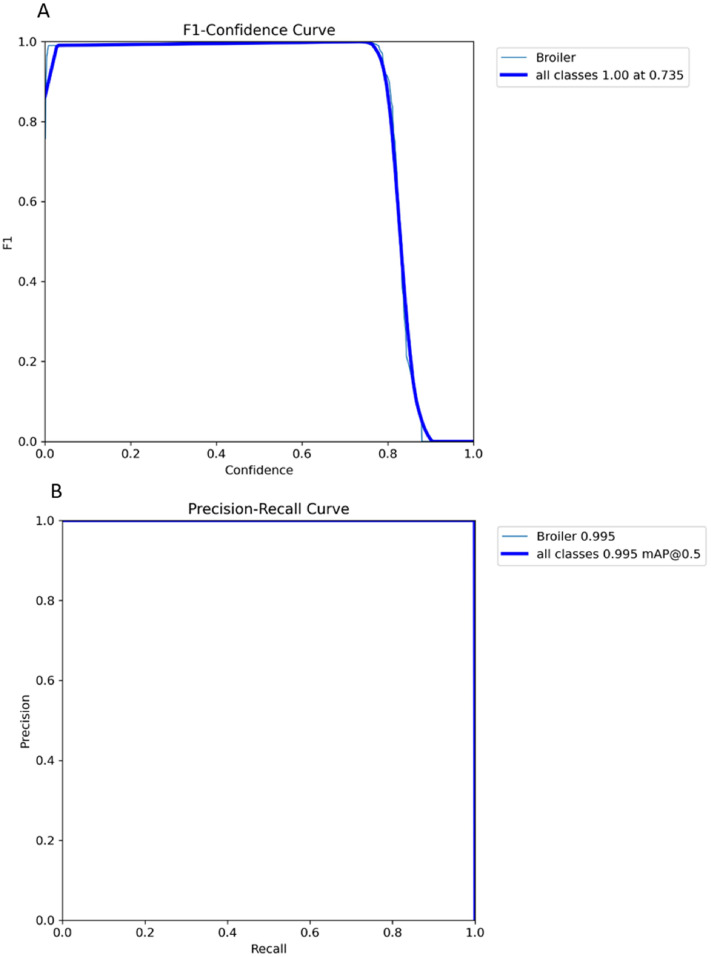
Model performance for bounding box and head keypoint detection: a) F1 score across various thresholds, b) precision-recall curve.

The model's performance for bounding box and head keypoint detection was evaluated using different metrics. The best model, based on performance on the validation set, achieved an mAP50-95 of 0.81 for bounding box detection and 0.99 for head keypoint detection. When evaluated on the test set, this model achieved the same mAP50-95 for both tasks. Fig. 6**a** illustrates the F1 score across various thresholds, providing insights into the model's balance between false positives and false negatives in the test set. Fig. 6**b** presents the precision-recall curve, depicting the trade-offs between precision and recall across varied thresholds. The model achieved a score of 0.99 at a 0.5 threshold for both tasks in the test set.

### The relationship of top-view features with walking ability and leg health

The median (interquartile range in parentheses) value of lateral body oscillations, step count, and completion time were 10.1 (3.6) px, 14 (5.8) steps, and 7.3 (4.7) seconds, respectively. All pairwise correlations between top-view features were significant and positive. The correlation of lateral body oscillations with step count was 0.29 (95 % confidence interval [**CI**]: 0.11-0.44, *P* = 0.002), and with completion time it was 0.22 (95 % CI: 0.04-0.39, *P* = 0.016). Completion time and step count were highly correlated (0.82, 95 % CI: 0.74-0.87, *P* < 0.001). The pairwise scatter plots of walking features are shown in **Supplementary Figure 1.**

Birds in different GS categories showed differences in their lateral body oscillation, completion time, and step count (Table 2). Birds with higher (i.e., worse) GS showed a tendency for higher lateral body oscillation levels, with no relationship between body weight and the level of body oscillation (*P* = 0.982). Higher GS were also associated with more steps and more time to complete the walkway test. Body weight was not related to either step count (*P* = 0.402) or completion time (*P* = 0.288).

**Table 2:** Top view features by gait, hock burn, and footpad dermatitis score categories^1^.

Feature	Gait score			P	Hock burn		P	Footpad dermatitis			P
	GS 1	GS 2	GS 3+		HB 0	HB 1		FPD 0	FPD 1	FPD 2	
Lateral body oscillations (px)	9.3^A^ (0.4)	10.7^B^ (0.5)	10.9^AB^ (1.0)	0.055	9.9 (0.3)	10.3 (1.0)	0.654	10.0 (0.4)	9.6 (0.5)	10.0 (0.8)	0.766
Step count (n)	12.6^a^ (0.5)	14.7^b^ (0.7)	22.4^c^ (1.4)	<0.001	14.1 (0.5)	12.7 (1.6)	0.374	14.1 (0.6)	14.2 (0.7)	13.5 (1.2)	0.890
Completion time (seconds)	7.0^a^ (0.6)	8.8^a^ (0.8)	21.6^b^ (1.6)	<0.001	8.9 (0.6)	6.4 (2.0)	0.225	8.7 (0.8)	8.6 (0.9)	9.1 (1.5)	0.962

1 Least squares means (standard errors in parentheses) ^a, b, c^ Groups not sharing a common superscript letter differ significantly (P-values adjusted using Tukey contrasts) ^A, B, C^ Groups not sharing a common superscript letter show a tendency for difference (P-values adjusted using Tukey contrasts)

As opposed to GS as a proxy for walking ability, we observed no differences in top-view features by HB or FPD categories as leg health indicators (Table 2). Body weight was also not significant in any of these models (all *P* > 0.26).

The confusion matrix of K-means clusters against gait score categories is shown in Table 3. Cluster A was characterized by the lowest lateral oscillation (8.49 ± 2.76, mean±SD), lowest number of steps (9.98 ± 2.08), and shortest completion time (4.68 ± 1.57 s). The lateral oscillation levels in cluster B (10.30 ± 2.80) were higher than in cluster A, and cluster B had the highest step counts (22.50 ± 3.91) and the longest completion times (22.20 ± 6.07 s), on average. Cluster C had similar, but numerically even higher lateral oscillation than cluster B, on average (11.10 ± 2.77), accompanied by intermediate step counts (15.7 ± 2.26) and completion times (9.15 ± 2.37). Most birds with GS1 were clustered into cluster A (52.1 %), with GS2 into cluster C (52.6 %), and GS3+ into cluster B (66.7 %). The distribution of broilers by gait score category and cluster is shown in **Supplementary Figure 2**.

**Table 3:** The distribution of birds by gait score category in the clusters identified via K-means clustering (% within gait score group in parentheses).

Cluster	Gait score group		
	GS 1 (n = 71)	GS 2 (n = 38)	GS 3+ (n = 9)
A (n = 50)	37 (52.1)	13 (34.2)	0 (0)
B (n = 13)	2 (2.8)	5 (13.2)	6 (66.7)
C (n = 55)	32 (45.1)	20 (52.6)	3 (33.3)

## Discussion

In this study, we examined whether and how automatically extracted top-view walking features of broilers walking in a corridor relate to manually determined walking ability and leg health scores. Birds that were manually categorized as having worse GS had longer estimated completion times, higher step counts, and a tendency for higher lateral body oscillation levels. The length-width ratio of the bounding box during walking, hypothesized to be a potential indicator of wing support while walking, was found not to be a suitable proxy. The top-view walking features were found not to be indicative of HB and FPD in broilers at the relatively low HB and FPD scores observed in our sample.

### Object detection model performance

The trained deep-learning model achieved good performance in broiler and head keypoint detection. We used YOLOv8, which supports multiple computer vision tasks such as object detection and keypoint detection within a single model framework, and simplifies the workflow and improves efficiency. The model was trained only on broilers within the walkway. As a result, the model did not detect broilers outside of this area, which sped up the process with not having the extra step to select the broilers in the region of interest (i.e., the walkway).

### Top-view features as proxy for gait

Birds with GS3+ showed longer estimated completion times in the corridor than birds with GS1 or GS2. This suggests that they either walked slower, sat down more in between, or showed a combination of both. This result aligns well with literature. For example, Li et al. (2023) observed higher average and maximum linear moving speeds in GS0 broilers than in GS1 or GS2 broilers, and Aydin et al. (2017a) observed lower speeds in GS3 and GS4 birds than in GS0, GS1 and GS2 birds. Pereira et al. (2021) also observed lower walking velocities for birds with higher GS, with GS0 and GS1 birds showing the highest velocities, GS4 and GS5 birds showing the lowest velocities, and GS2 and GS3 birds in between. In terms of resting more in between walking bouts, Aydin et al. (2017b) studied broilers walking in a corridor, using a 3D camera, and observed an increase in number of lying events from GS0 and GS1 (which did not differ from each other) to GS2, GS3 and GS4 birds. These observations seem to align well with the differences in completion time observed here. It is important to note that the birds were sometimes nudged to move through the corridor in this study, and whether the birds were nudged and how quickly this would be done was not fixed. This may have impacted the completion time. However, it is expected that nudging the birds (especially the slower ones) to move would theoretically bring the completion times closer together, potentially masking any differences in completion time between the GS groups. Given that a difference in completion time was still observed here suggests that the differences in completion time between birds with different GS were quite pronounced. It has been suggested that reductions in movement speed can contribute to reducing peak vertical forces and stress on the musculoskeletal system (Corr et al., 2007). Broilers that received a non-steroidal anti-inflammatory drug showed higher velocities than before the treatment (Caplen et al., 2013), suggesting that slower walking is a potential response to pain.

Birds with higher GS furthermore showed a higher step count, i.e., number of steps to complete the walkway. Aydin (2017a) also observed a higher step frequency for birds with higher GS, where GS3 and GS4 birds had higher step frequencies than GS0, GS1 and GS2 birds, and GS4 birds also showed a higher step frequency than GS3 birds. In the current study, this difference in step count was already detectable for lower GS, i.e., also between birds with GS1 or GS2. Caplen et al. (2012) observed, when comparing GS0 and GS3 birds, that GS3 birds had shorter stride durations, shorter stride lengths, and lower vertical leg displacement (i.e., step height). These observations align well with the current study: if lame broilers make short steps, they require more steps to complete the walkway. A lower step height for birds with worse GS was also observed in our previous study (Fodor et al., 2023) and might be linked to the positive correlation between the vertical leg displacement and stride length that was observed by Caplen et al. (2012).

In addition, birds with higher GS showed a tendency for higher lateral body oscillation levels, indicating more swinging during walking. This observation is in line with other studies (e.g., Caplen et al., 2012; Aydin, 2017a). However, Li et al. (2023) reported opposite results, with more lateral body oscillation being observed in broilers with GS0 and GS1 than in birds with GS2, but they note that this may have been a consequence of their GS2 birds sitting down or waddling instead of walking upright in their study. It has been reported that broilers move their center of gravity laterally towards the supporting leg during walking (Reiter & Bessei, 1997). In earlier work, we have observed that broilers with GS > 2 have a smaller hock-feet distance ratio, suggesting that the feet of these birds were relatively more spread out than the hocks compared to broilers with GS ≤ 2 (Fodor et al., 2023). If broilers with worse GS place their feet more outwards during walking and they move their center of gravity towards the supporting leg, this might explain the higher levels of lateral body oscillation observed for these birds.

In our study, even when combining only three walking features (two of which were strongly correlated), a clustering algorithm showed potential to distinguish different GS categories, though the separation between GS1 and GS2 was less clear. This aligns with Aydin (2017a), who concluded that changes in the separate walking features were detectable from GS3 onwards. By developing further features that provide a more detailed description of different aspects of walking characteristics, top-view computer vision may capture information for more sophisticated and accurate classification of walking ability, also picking up on more subtle changes to better distinguish lower GS categories.

In this study, we used a wire-net panel attached to a stick to gently stimulate birds that did not progress through the corridor. Stimulating broilers to walk may influence certain walking characteristics. For example, Almeida Paz et al. (2019) found higher walking speed across all gait score categories in birds that were stimulated using sound during gait score testing, suggesting that discomfort due to gait abnormalities may be suppressed when stimulated to walk. However, even with stimulation, birds in higher gait score categories still had lower average walking speeds compared to unstimulated birds with better gait scores. As stimulation can be difficult to avoid in practice during a walkway test, we suggest to develop and report a protocol for the stimulation of the birds in future studies.

### Top-view features as proxy for leg health

Severe FPD lesions may negatively affect the gait of birds (Tullo et al., 2017). Furthermore, birds with poor gait might show lower locomotor activity levels than birds with a good gait (e.g., Van Hertem et al., 2018; van der Sluis et al., 2021), which could increase the risk of HB due to increasing contact of the hocks with the litter (suggested by e.g. Haslam et al., 2007; de Jong et al., 2014). However, none of the top-view walking features in this study were found to be indicative of HB or FPD in broilers. We furthermore observed no statistically significant associations between gait, HB and FPD scores. This contrasts with observations by for example Granquist et al. (2019), who found that higher GS were associated with higher HB and FPD scores. However, it must be noted that in our study most birds showed no signs of HB or FPD and only a few birds showed mild cases of HB and FPD, which might not have been severe enough to affect walking ability or top-view walking characteristics.

### Towards practical implementation

In this study, we examined broilers' top-view walking characteristics using a walkway, accounting for movement direction by fitting a path for each bird and measuring deviations from this path as lateral body oscillation. This approach allowed us to estimate lateral body oscillation even when birds walked curved paths, which will probably occur under practical circumstances. While further improvements are likely needed to refine movement trajectory modeling for practical applications, this study highlights the potential of using deviations from this trajectory as a walking feature in home pens where voluntary walking can be tracked without handling or disturbing the birds. While this method offers potential to assess walking ability, challenges remain with individual bird identification in group-housed settings.

Overall, the results of this study indicate that top-view video recordings can provide insight into birds’ walking ability, using features related to movement speed, step count and lateral body oscillation. However, the top-view walking characteristics do not provide clear insight into leg health aspects (HB and FPD) if the lesions are relatively mild.

## Declaration of competing interest

The authors declare the following financial interests/personal relationships which may be considered as potential competing interests: Cobb Europe was involved in the study design and in the collection of data. The funders had no role in the decision to submit the article for publication.

## Appendix Supplementary materials

Image, application 1
