# Supplementary material for: Top-view characterization of broiler walking ability and leg health using computer vision
Source: Poult Sci. 2024 Dec 22;104(2):104724. doi: 10.1016/j.psj.2024.104724 (PMC11761931; doi:10.1016/j.psj.2024.104724)
Supplement: Supplementary file 1 [file mmc1.docx]

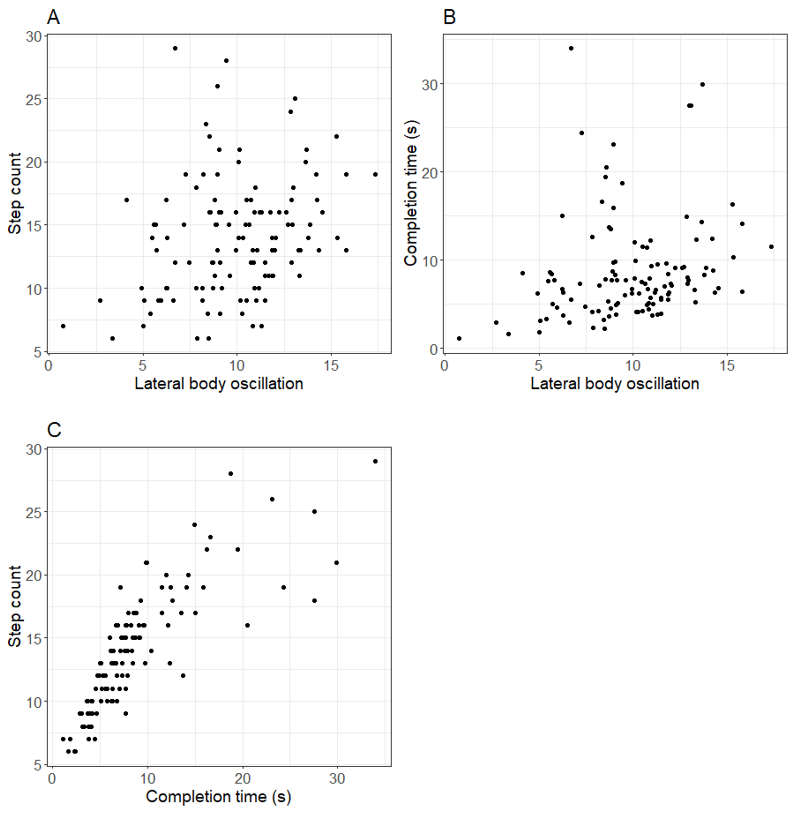


**Supplementary Figure 1.** Scatter plots of walking features, (A) lateral body oscillation vs. step count (r = 0.29), (B) lateral body oscillation vs. completion time (r = 0.22), (C) completion time vs. step count (r = 0.82)


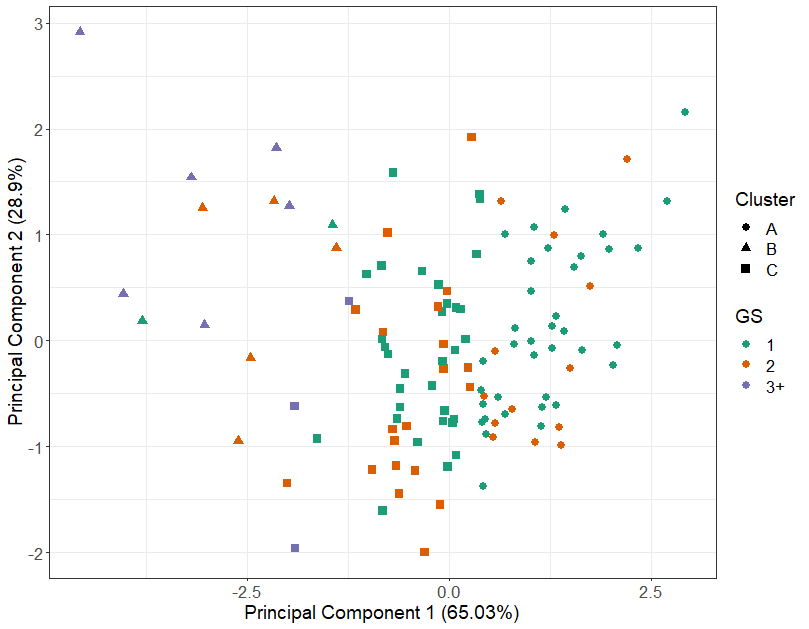


**Supplementary Figure 2.** The distribution of broilers by gait score category (colours) and cluster (shapes) plotted on the first two principal components.
